# Individualized Radiation Dose Assessment in Low-Dose Chest CT: The Role of DLPss and Topogram Optimization

**DOI:** 10.3390/jcm15093474

**Published:** 2026-05-01

**Authors:** Arkadiusz Szarmach, Dominika Sabiniewicz-Ziajka, Małgorzata Grzywińska, Paweł Gać, Marcel Zoch, Maciej Piskunowicz, Magdalena Wszędybył-Winklewska

**Affiliations:** 1 2nd Department of Radiology, Medical University of Gdansk, 80-210 Gdansk, Poland; dominika.sabiniewicz@gumed.edu.pl; 2 Neuroinformatics and Artificial Intelligence Laboratory, Department of Neurophysiology, Neuropsychology and Neuroinformatics, Medical University of Gdansk, 80-210 Gdansk, Poland; malgorzata.grzywinska@gumed.edu.pl; 3 Centre for Diagnostic Imaging, 4th Military Hospital, 50-981 Wroclaw, Poland; pawelgac@interia.pl; 4 Department of Population Health, Division of Environmental Health and Occupational Medicine, Wroclaw Medical University, 50-368 Wroclaw, Poland; 5 Students’ Scientific Association, Faculty of Medicine Ludwik Rydygier Collegium Medicum, Nicolaus Copernicus University, 85-067 Bydgoszcz, Poland; marcelzoch76@gmail.com; 6 1st Department of Radiology, Medical University of Gdansk, 80-210 Gdansk, Poland; maciej.piskunowicz@gumed.edu.pl; 7 Institute of Health Sciences, Pomeranian University, 76-200 Slupsk, Poland

**Keywords:** low-dose computed tomography, radiation dose, size-specific dose–length product (DLPss), topogram, radiation optimization, deep learning reconstruction

## Abstract

**Background:** The increasing use of computed tomography (CT) has led to a substantial rise in population exposure to ionizing radiation, highlighting the need for accurate and individualized dose assessment methods. This study aimed to evaluate a novel dosimetric parameter—the size-specific dose–length product (DLPss)—in low-dose chest CT (LDCT) protocols and to compare its performance with conventional dose metrics. **Methods:** A retrospective single-center analysis was conducted in a cohort of 221 patients undergoing LDCT of the chest. Anthropometric parameters were used to calculate the size-specific conversion factor (*k*), enabling determination of SSDE and DLPss. Dose parameters (CTDIvol, DLP, SSDE, and DLPss) were analyzed and compared with data from a standard chest CT cohort (*n* = 134) from the first study in the series. The contribution of the topogram to total radiation dose was also assessed. All examinations were considered diagnostically adequate in routine clinical evaluations. **Results:** The mean CTDIvol in the LDCT group was 1.33 mGy, with a DLPss of 61.93 mGy·cm and an estimated effective dose below 0.7 mSv, representing a dose reduction exceeding 82% compared to standard CT. DLPss values were approximately 23% higher than conventional DLP, indicating underestimation of dose by standard metrics. The topogram accounted for 10.23% of total radiation dose in LDCT, significantly higher than in standard CT (1.84%). Significant sex-related differences were observed in CTDIvol, DLP, and DLPss, but not in SSDE. **Conclusions:** DLPss provides a more comprehensive and individualized assessment of radiation exposure than conventional dose metrics by integrating patient size and scan length. The substantial contribution of the topogram to total dose in LDCT highlights the need for its optimization, particularly in long-term screening programs. From a clinical perspective, implementation of DLPss may improve patient-specific risk stratification and support more precise monitoring of cumulative radiation exposure, especially in populations undergoing repeated imaging, such as lung cancer screening cohorts. Advanced reconstruction algorithms, including deep learning-based methods, may enable further dose reductions and warrant future clinical investigation.

## 1. Introduction

Computed tomography (CT) is one of the most widely used and rapidly evolving diagnostic imaging modalities [1]. According to the United Nations Scientific Committee on the Effects of Atomic Radiation (UNSCEAR), CT accounts for more than 62% of the collective dose of ionizing radiation from medical sources, despite representing only approximately 10% of all imaging procedures [2]. One of the most important strategies for reducing this exposure is low-dose computed tomography (LDCT), proven effective as a lung cancer screening method in the National Lung Screening Trial (NLST) [3]. Furthermore, the OPTIMACT study demonstrated that ultra-low-dose computed tomography (ULDCT) achieved higher diagnostic accuracy than standard-dose CT in acute pulmonary conditions, with a median radiation dose of only 0.2 mSv [4].

The clinical applications of LDCT also include the assessment of vascular calcifications [5,6], the evaluation of pulmonary emphysema [7,8], and the monitoring of patients with chronic obstructive pulmonary disease (COPD) [9]. The European Society of Thoracic Imaging (ESTI) and the National Comprehensive Cancer Network (NCCN) recommend LDCT as the method of choice for lung cancer screening in high-risk populations [10,11].

Alongside the expanding clinical indications for low-dose CT, there has been rapid technological progress in CT image reconstruction algorithms, which have become a key tool enabling dose reduction. Deep learning-based image reconstruction (DLIR) reduces image noise while preserving natural texture and improving spatial resolution, and has been shown to enhance the detectability of pulmonary nodules in ultra-low-dose examinations [12].

However, precise assessment of the individual radiation dose absorbed by the patient remains a methodological challenge. Commonly used radiation exposure metrics, such as CTDIvol (CT dose index volume), DLP (dose–length product), and ED (effective dose), are derived from standardized phantoms and do not account for actual patient body size [13]. A partial solution to this limitation is the size-specific dose estimate (SSDE), calculated as the product of CTDIvol and a variable conversion factor (k) determined based on the patient’s effective body diameter.

These limitations inspired our research group to propose a novel parameter—DLPss (size-specific dose–length product), which integrates patient body dimensions with the actual scanned length. This parameter has been previously introduced and validated by our team in standard CT examinations of the chest [14] and abdomen [15], demonstrating higher precision in estimating individual radiation exposure compared to conventional DLP.

A particularly underappreciated dosimetric aspect of low-dose protocols is the contribution of the topogram to the total radiation dose. In standard CT protocols, its contribution is negligible [14]. However, in LDCT protocols, where the diagnostic scan dose is substantially reduced, the topogram may account for several percent up to nearly 40% of the total examination dose [16]. The present study—the third in an institutional series—makes three original scientific contributions beyond confirmatory application of DLPss: (1) it provides the first large-cohort, real-world clinical quantification of the topogram’s relative dose contribution in LDCT (10.23%), a value markedly higher than theoretically derived estimates and with direct implications for lifetime dose management in screening programs; (2) it demonstrates the cross-protocol robustness of DLPss by confirming that the parameter systematically exceeds DLP by ~23% across the full clinical dose spectrum, from standard to low-dose protocols; and (3) it replicates, for the first time in an LDCT cohort, the finding that size-correction via the k-factor attenuates sex-related dose differences, strengthening the case for individualized dosimetry in population-based screening.

The aim of this study was to evaluate CTDIvol, SSDE, DLP, and DLPss in 221 low-dose chest CT examinations, with particular emphasis on: (1) the contribution of the topogram to total radiation dose and its implications for cumulative lifetime exposure, (2) sex-related differences in dosimetric parameters, and (3) comparison with a standard-dose chest CT cohort from a previous study in the series [14].

## 2. Materials and Methods

### 2.1. Study Design, Population, and Ethical Considerations

This was a retrospective, single-center study. The study protocol was approved by the appropriate institutional ethics committee, and all included patients provided informed consent. The inclusion and exclusion criteria were identical to those applied in previous studies in this series [14,15].

The study cohort comprised 221 low-dose chest CT examinations performed in 221 different adult patients (135 men and 86 women). LDCT examinations were identified from the CT registry using a dedicated search tool (MedStream Designer, Transition Technologies Science Ltd., Warsaw, Poland, 2015).

The results were compared with a cohort of 134 patients (66 men and 68 women) who underwent standard-dose chest CT, previously analyzed in the first study of the series [14]. The methodology for determining all dosimetric parameters, including body size measurements, calculation of effective diameter, determination of the conversion factor k, and calculation of SSDE and DLPss, was identical to that used in previous studies in this series [14,15], enabling direct and reliable comparison of the results.

### 2.2. CT Acquisition Protocol

All examinations were performed using a multidetector CT scanner (SOMATOM Definition Flash, Siemens Healthineers, Erlangen, Germany) with a dedicated low-dose chest protocol. Image acquisition was conducted in the craniocaudal direction during breath-hold at full inspiration.

In all examinations, automatic tube current modulation (TCM; CARE Dose4D, Siemens Healthineers) was applied, allowing adjustment of exposure parameters according to patient body size and habitus. In all examinations, topogram acquisition followed a fixed institutional protocol with standardized parameters applied uniformly across all patients regardless of body habitus; no manual adjustment of topogram kVp or mAs based on patient size was performed. A standard topogram was acquired prior to the diagnostic scan.

### 2.3. Anthropometric Measurements and Dose Calculations

For each patient, body weight and height were recorded, and body mass index (BMI) was calculated according to the formula:

**BMI = weight (kg)/height^2^ (m)** (1)

The lateral chest dimension (A) and the anteroposterior dimension (B) were measured directly on the topogram at the mid-scan level, in accordance with the methodology used in previous studies in this series [14,15]. All measurements were performed by a single experienced radiologist, following the standardized protocol established in the first study of this series [14], ensuring methodological consistency across the three publications. The effective diameter (Deff) was calculated as the square root of the product of A and B:

(2) $$Deff\;=\;\;\sqrt{AP\;\times \;LAT}$$

Based on the calculated effective diameter, a variable conversion factor ***k*** was determined using tables published by the American Association of Physicists in Medicine (AAPM) in Report No. 204 [17]. The k factor, dependent on patient body size, was used to calculate the size-specific dose estimate (SSDE) according to the formula:

**SSDE = CTDIvol × *k*** (3)

and to determine the novel parameter DLPss (size-specific dose–length product), first introduced by our group in a previous study [14]:

**DLPss = DLP × *k*** (4)

The effective dose (ED) was estimated using a constant conversion coefficient ***ƒ*** (0.014 mSv/mGy·cm), established for adult chest CT examinations in AAPM Report No. 96 [18], according to the formula:

**ED = DLP × *f*** (5)

Other parameters, including CTDIvol, DLP, and acquisition length (L), were obtained separately for the topogram and for the diagnostic scan from the scanner-generated dose report. The results were compared with corresponding data from the standard-dose chest CT group reported in the first study of the series [14].

### 2.4. Statistical Analysis

Statistical analysis was performed using Statistica software (version 13; TIBCO Software Inc., Palo Alto, CA, USA). Continuous variables were expressed as mean ± standard deviation (SD) with range (minimum–maximum). Normality of distribution was assessed using the Shapiro–Wilk test. Differences between sex groups were evaluated using the Mann–Whitney U test or Student’s *t*-test, depending on data distribution. A *p*-value < 0.05 was considered statistically significant.

## 3. Results

### 3.1. Study Population Characteristics

A total of 221 low-dose chest CT examinations were analyzed in 221 patients (135 men and 86 women). The mean age of the study population was 58.47 ± 15.07 years (range: 31.22–77.92 years). Statistically significant sex-related differences were observed for all anthropometric parameters.

The calculated effective chest diameter was 29.41 ± 2.80 cm (range: 24.07–33.71 cm), and the corresponding variable conversion factor k was 1.26 ± 0.13 (range: 1.06–1.53; *p* < 0.001 for women vs. men), reflecting substantial heterogeneity in anatomical dimensions within the study group. A detailed summary of all analyzed parameters is presented in Table 1.

**Figure 1:**
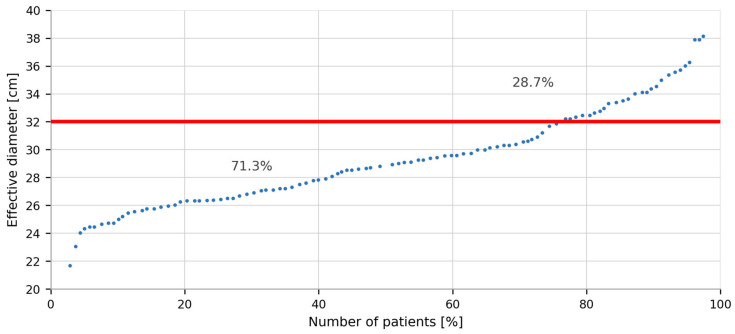
Percentage distribution of patients according to effective diameter (Deff) relative to the reference chest phantom diameter (32 cm).

In the study population, nearly 71% of patients had a Deff below the 32 cm phantom reference, confirming that CTDIvol and DLP systematically overestimate dose in the majority of patients when size correction is not applied—and underestimate it in the remaining 29% with larger body habitus. This distribution directly motivates the clinical necessity of the individualized *k*-factor correction and the DLPss parameter (Figure 1).

### 3.2. Diagnostic Scan Dose Parameters and Size-Corrected Metrics

The mean CTDIvol for the diagnostic acquisition was 1.33 ± 0.38 mGy (range: 0.72–2.14 mGy). The SSDE, calculated as the product of CTDIvol and the variable conversion factor k, was 1.64 ± 0.33 mGy (range: 1.10–2.44 mGy).

The mean DLP was 50.35 ± 15.93 mGy·cm (range: 24.00–86.00 mGy·cm), while the mean DLPss was 61.93 ± 14.62 mGy·cm (range: 36.72–98.04 mGy·cm), exceeding the conventional DLP by approximately 23%.

Statistically significant sex-related differences were observed for CTDIvol (men: 1.55 vs. women: 1.12 mGy; *p* = 0.007), DLP (men: 60.1 vs. women: 40.6 mGy·cm; *p* = 0.003), and DLPss (*p* = 0.037). The difference in SSDE did not reach statistical significance (*p* = 0.096).

### 3.3. Comparison with the Standard-Dose Chest CT Group

All analyzed dose parameters in the LDCT group were significantly lower than the corresponding values in the standard-dose chest CT group reported in the first study of the series [14] (*p* < 0.001 for all comparisons).

CTDIvol accounted for 17.0%, SSDE for 17.5%, DLP for 17.6%, and DLPss for 18.0% of the corresponding values in the standard-dose group (Table 2), reflecting a dose reduction exceeding 82% across all parameters.

Notably, the magnitude of dose reduction was comparable for both size-corrected (SSDE, DLPss) and non-size-corrected (CTDIvol, DLP) parameters, reflecting similar anthropometric characteristics between the two study groups.

### 3.4. Topogram Dose and Its Contribution to Total Examination Dose

The mean CTDIvol of the topogram was 0.14 ± 0.01 mGy (range: 0.10–0.14 mGy), and the mean DLP of the topogram was 5.15 ± 0.59 mGy·cm (range: 4.00–6.00 mGy·cm). No statistically significant sex-related differences were observed for any topogram-related parameters, including topogram length (*p* = 0.152), CTDIvol (*p* = 0.331), or DLP (*p* = 0.264).

The DLP of the topogram was comparable to that observed in standard-dose chest CT in the first study of the series (5.28 mGy·cm) [14]. However, due to the substantial reduction in diagnostic scan dose in the LDCT protocol, the relative contribution of the topogram to total dose increased to 10.23%, representing more than a fivefold increase compared to standard-dose chest CT (1.84%) [14].

The mean length of the diagnostic scan in the low-dose protocol was 403.00 ± 29.84 mm, while the mean topogram length was 386.55 ± 50.19 mm; the difference (ΔL = 21.95 ± 40.84 mm) was not statistically significant (*p* = 0.631).

## 4. Discussion

The present study constitutes the third part of a series investigating the feasibility of precise assessment of ionizing radiation dose in computed tomography and represents a direct evaluation of a novel dosimetric parameter proposed by our group, the size-specific dose–length product (DLPss), this time within low-dose protocols. The increasing number of CT examinations and the associated high cumulative radiation dose result in CT contributing the largest proportion to population exposure [19,20]. In the context of millions of examinations performed annually, these factors clearly indicate the need for reliable methods of dose monitoring and reduction. The present study makes three original contributions beyond confirmatory application of the DLPss formula: the first real-world clinical quantification of the topogram contribution in LDCT (10.23%); demonstration of cross-protocol robustness of DLPss across the full dose spectrum; and first replication in an LDCT cohort of the k-factor’s sex-difference attenuating effect. DLPss systematically exceeds DLP by ~23% (61.93 vs. 50.35 mGy·cm), providing substantially higher and more individualized radiation risk estimates in both standard [14,15] and—as demonstrated here for the first time—low-dose protocols. It is important to distinguish between DLPss and the effective dose (ED): while ED, calculated using the fixed population-level coefficient *f* = 0.014 mSv/mGy·cm from AAPM Report 96, is appropriate for epidemiological comparisons and population-level benchmarking, it does not reflect individual patient anatomy. DLPss, by contrast, is the appropriate metric for individualized dose monitoring and cumulative lifetime dose tracking in screening programs. Both metrics serve distinct and complementary roles in clinical dosimetry.

The actual absorbed dose depends on several factors, including acquisition parameters, examination protocol, scanner type, and, most importantly, patient size and body habitus. Conventional dose metrics (CTDIvol and DLP) are derived using standardized phantoms with fixed reference diameters and therefore do not account for individual patient dimensions [21]. The DLP value approximates the true dose only when the patient’s effective diameter corresponds to that of the reference phantom (32 cm for the chest), which rarely occurs in clinical practice. In our cohort, the mean effective chest diameter (Deff) was 29.41 ± 2.80 cm, with 71.1% of patients presenting values below the reference phantom diameter (Figure 1). Similarly, in the standard chest CT group (first study in the series [14]), these values were 29.92 ± 3.45 cm and 69.9%, respectively, resulting in a mean CTDIvol nearly 20% higher than the scanner-reported value. This phenomenon arises directly from automatic tube current modulation (TCM), which adjusts exposure based on tissue attenuation through a feedback mechanism, leading to a logarithmic increase in tube current with increasing patient size [22]. Consequently, CTDIvol systematically underestimates exposure in larger patients and overestimates it in smaller individuals [23].

Although SSDE represents a significant advancement in radiation dose assessment, it remains subject to a fundamental methodological limitation: it describes the absorbed dose in a single selected cross-sectional slice rather than across the entire scan range [24]. This implies that for examinations performed with varying scan lengths (in our LDCT study ranging from 341 to 445 mm), SSDE does not reflect the true cumulative patient exposure, and SSDE values obtained in different examinations of the same patient may be comparable despite differences in total absorbed dose proportional to scan length. In contrast, DLPss, calculated as the product of DLP and a size-specific conversion factor (k), integrates patient body dimensions with actual scan length, providing an exposure estimate for the entire examination rather than a single slice. This distinction is clinically relevant both for comparisons between patients of different body habitus and in long-term screening programs, where variability in scan length between examinations may lead to substantial differences in total radiation exposure not captured by SSDE alone. Thus, DLPss combines the advantages of SSDE (patient size adjustment) with those of DLP (scan length inclusion), representing a more comprehensive parameter for individual radiation exposure assessment.

A key finding of this study is the objective quantification of the topogram contribution to total dose in low-dose examinations at 10.23%, along with its direct comparison to the standard-dose chest CT group from the same institution (1.84%) [14]. The mechanism underlying this discrepancy is clear: the absolute DLP of the topogram was nearly identical in both groups (5.15 vs. 5.28 mGy·cm), while the more than fivefold relative difference results solely from the substantial reduction in diagnostic scan DLP in the LDCT protocol, with topogram parameters remaining unchanged. In our institution, topogram acquisition followed a fixed protocol with standardized kVp and mAs applied uniformly across all patients, regardless of body habitus—no size-adaptive topogram optimization was performed. This observation highlights an underutilized opportunity: individualized topogram parameter adjustment, analogous to the TCM applied to diagnostic scans, could meaningfully reduce the topogram’s contribution to cumulative lifetime dose in screening programs. In the standard chest CT group from the first study in the series, the diagnostic scan length exceeded the topogram length by an average of 13.33 mm [14]. In the present LDCT cohort, this difference was 21.95 mm (*p* = 0.631) and similarly not statistically significant. This demonstrates that topogram length reliably reflects the planned scan range, and that adjustments to diagnostic scan length have relatively lower dosimetric impact compared to optimization of the topogram itself, consistent with observations by Cohen et al. and Campbell et al. [25,26]. In our LDCT cohort, the diagnostic range was shortened in nearly 30% of scans and extended in approximately 70%, consistent with previous findings [14]. From a clinical standpoint, this finding is particularly relevant in repeated imaging scenarios, where even small per-examination contributions may translate into a substantial cumulative dose.

Zanca et al. [27] demonstrated that unjustified extension of the scan range increased the effective dose of chest CT from 4.2 to 4.8 mSv, resulting in a 99% increase in thyroid dose and up to a 163% increase in breast dose. This observation is particularly relevant in the context of long-term screening programs. As emphasized by Vonder et al. [28], patients participating in long-term lung cancer screening may undergo more than 25 LDCT examinations during their lifetime. Assuming, based on our findings, a consistent topogram contribution of approximately 10%, its cumulative impact after 25 examinations would correspond to nearly 2.6 full diagnostic scans, a finding with direct clinical relevance. Schmidt et al. [16], based on phantom data and/or theoretical estimates, reported a topogram contribution ranging from 4% to 38%. Our study provides a concrete, clinically relevant value derived from real-world radiological practice. This finding justifies applying the same optimization strategies to the topogram as to the diagnostic scan, including reduction in tube current, limitation of scan length to the minimum necessary, and planning based on prior imaging or artificial intelligence algorithms. This highlights the need for incorporating individualized dosimetric parameters such as DLPss into longitudinal patient monitoring frameworks.

In the studied cohort, all four analyzed dose parameters in the LDCT group represented 17.0–18.0% of the corresponding standard CT values (CTDIvol: 17.0%, SSDE: 17.5%, DLP: 17.6%, DLPss: 18.0%), corresponding to an overall dose reduction exceeding 82%. This confirms the effectiveness of the applied LDCT protocol and is consistent with the literature. Diederich and Lenzen [29] demonstrated that using low tube current (25 mA) with high pitch (2) enables effective doses of 0.3 mSv in men and 0.55 mSv in women, corresponding to approximately 1.3 and 2.2 standard chest radiographs, respectively. In a large study by Larke et al. involving NLST participants, the mean CTDIvol was 2.9 mGy with an effective dose of 1.4 mSv [30]. Notably, the CTDIvol in our cohort (1.33 mGy) is more than twofold lower than that reported in the NLST, reflecting substantial progress in LDCT optimization over the past decade, including routine implementation of TCM, which was not required in the NLST protocol. For comparison, in the standard chest CT group from the first study in the series, the mean effective dose was 4.01 ± 1.4 mSv (3.62 ± 1.39 mSv for women and 4.41 ± 1.3 mSv for men) [14]. These values are consistent with large population-based studies reporting mean effective doses of 5.1–9 mSv for chest CT [31,32]. Compared with the present study, where DLPss was 61.93 mGy·cm and estimated effective dose was only 0.7 mSv, this clearly illustrates the magnitude of radiation reduction achieved with low-dose acquisition protocols.

An important tool for assessing and comparing dosimetric parameters is the diagnostic reference level (DRL). Introduced in 1996 in response to significant inter-institutional and international variability in radiation doses for identical scanning protocols [33,34], DRLs are recommended by the International Atomic Energy Agency (IAEA) and the International Commission on Radiological Protection (ICRP) as optimization tools, defined as the 75th percentile of dose distributions [35,36]. The results of the present study (CTDIvol 1.33 mGy, DLPss 61.93 mGy·cm) place the applied LDCT protocol well below reference levels for standard chest CT, which serves as the most widely available benchmark, confirming its high effectiveness in real-world dose reduction.

Alongside advancements in protocol optimization, there has been rapid development of CT image reconstruction algorithms, offering further potential for dose reduction while maintaining diagnostic quality. Traditional filtered back projection (FBP) produces increasing image noise at low doses [37], whereas iterative reconstruction (IR) techniques enable dose reductions of 27–54% compared to FBP [38]. A major breakthrough has been the introduction of deep learning-based image reconstruction (DLIR), which more effectively reduces noise while preserving natural image texture [39]. Zellner et al. [40], in a phantom study, demonstrated that diagnostic-quality chest CT images can be achieved at extremely low dose levels; however, these findings require clinical validation. Jiang et al. [12] showed that DLIR reduces image noise by 21% compared to iterative methods in ULDCT while improving pulmonary nodule detectability. Yeom et al. [41] confirmed the reliability of ULDCT with DLIR-low for emphysema quantification at a markedly reduced dose. Shiri et al. [42] and Mikayama et al. [43] further demonstrated the potential and limitations of deep learning approaches in ultra-low-dose imaging, particularly in the evaluation of ground-glass opacities. In addition, Frings et al. [44] showed that photon-counting CT enables further dose reduction with improved image quality. Importantly, DLPss does not require recalibration for DLIR or any other reconstruction algorithm, because it is a pre-reconstruction, physics-based dose metric: DLPss = DLP × k quantifies radiation energy deposited in patient tissues based on tube output and patient anatomy, independent of how the acquired signal is subsequently processed. DLIR algorithms reduce perceived image noise post-acquisition without altering the physical dose delivered.

Sex-dependent differences observed in CTDIvol, DLP, and DLPss are consistent with prior series studies [14,15] and reflect TCM responding to tissue attenuation. The non-significant difference in SSDE (*p* = 0.096) confirms that *k*-factor correction partially normalizes sex-related dose disparities, as previously shown in standard chest CT [14]. We additionally note the feasibility and desirability of automating DLPss calculation within clinical dose management systems. Modern CT dose management platforms (e.g., Radimetrics, DoseWatch) already extract CTDIvol and DLP automatically from DICOM headers. Effective diameter can in principle be computed automatically from topogram pixel data using body segmentation algorithms, several of which have been validated in the literature. Integration of automated Deff calculation and k-table lookup into existing dose management software would render DLPss a zero-overhead parameter at the point of care, removing the manual measurement barrier in high-volume screening centers.

This study has several limitations. The analysis is retrospective and single-center, which may limit direct generalizability to centers using different scanner models or reconstruction algorithms; however, performing all examinations on the same device ensures internal data consistency. Regarding the k-factor calculation: the use of a single mid-scan cross-sectional measurement to derive the conversion factor k is a methodological simplification inherent to all current AAPM Report 204-based SSDE approaches. In patients with significant anatomical variation along the craniocaudal axis—such as those with advanced pulmonary emphysema or abdominal obesity extending into the scan field—this single-level approach may introduce systematic error: k derived at the mid-scan level may underestimate dose in the cranial lung apices (typically smaller cross-sections) or overestimate it in larger subdiaphragmatic regions. We did not prospectively collect multi-level measurements, and a formal sensitivity analysis cannot be performed on the current dataset. Future prospective studies should employ multi-level or fully automated Deff measurements to quantify this uncertainty. All 221 examinations were used in routine clinical care and interpreted as diagnostically adequate by subspecialty-trained thoracic radiologists—confirming real-world diagnostic utility. However, structured image quality assessment using standardized instruments (validated Likert scales, objective noise metrics such as SNR and CNR, or nodule detectability scores) was not part of the original data collection protocol. This represents a methodological limitation: routine clinical reporting, while affirming practical diagnostic utility, does not formally establish that image quality is preserved across the full range of dose levels observed in our cohort, nor does it enable dose–quality trade-off analysis. In a study reporting >82% dose reduction, the absence of objective image quality data means that our findings should be interpreted as characterizing the dosimetric profile of an established clinical protocol, rather than as a non-inferiority demonstration for image quality. Similarly, all body dimension measurements were performed by a single observer; inter-observer variability data are not available for this dataset. These gaps define the primary objectives of a planned prospective follow-up study within this series, which will combine routine DLPss assessment with prospective structured image quality scoring, multi-observer measurements with ICC analysis, and evaluation of DLIR-enabled dose reduction.

## 5. Conclusions

The present study confirms the effectiveness of currently applied LDCT chest protocols in reducing exposure to ionizing radiation. The k-factor, determined individually for each patient based on the effective chest diameter, enables the calculation of SSDE and DLPss, parameters that provide a more comprehensive and individualized assessment of radiation exposure compared to conventional CTDIvol and DLP. The implementation of DLPss, a novel parameter proposed by our group and applied for the first time in LDCT protocols, represents an important step toward more precise monitoring of patients’ lifetime radiation dose.

Particular attention should be paid to the high relative contribution of the topogram to the total dose in LDCT examinations. This results from a fundamental asymmetry between the reduction in the diagnostic scan dose and the fixed parameters of the topogram, and becomes clinically relevant particularly in the context of long-term screening programs. Topogram optimization should therefore be considered an integral component of dose reduction strategies in LDCT rather than a neglected element of the procedure. Systematic sex-related differences were also observed in diagnostic scan dose parameters, with no such differences in topogram parameters, which is consistent with the mechanism of automatic tube current modulation and findings from previous studies in this series.

Deep learning-based reconstruction algorithms offer the potential for further substantial dose reduction without compromising diagnostic image quality. Clinical validation of these technologies, combined with prospective assessment of SSDE and DLPss, multi-observer measurement protocols, and structured image quality scoring using validated scales, should form the primary objectives of future work in this series. Automation of DLPss calculation within clinical dose management systems represents a near-term, achievable step toward routine individualized dose monitoring in high-volume LDCT screening programs.

## Figures and Tables

**Table 1:** Anthropometric and dosimetric characteristics of the study population (*n* = 221; 135 men, 86 women). SD—standard deviation; F—women; M—men.

Parameter	Mean ± SD	Min	Max	SD	*p* (F/M)
**A. Anthropometric and Patient Characteristics**					
Age (years)	58.47 ± 15.07	31.22	77.92	15.07	0.021
Body weight (kg)	72.70 ± 17.06	52.00	106.00	17.06	<0.001
Height (cm)	168.15 ± 11.38	152.00	193.00	11.38	<0.001
BMI (kg/m^2^)	25.41 ± 3.68	19.00	32.00	3.68	0.004
Lateral dimension A (cm)	34.06 ± 3.36	26.47	39.78	3.36	<0.001
AP dimension B (cm)	25.43 ± 2.63	21.56	31.05	2.63	<0.001
Effective diameter Deff (cm)	29.41 ± 2.80	24.07	33.71	2.80	<0.001
Conversion factor k	1.26 ± 0.13	1.06	1.53	0.13	<0.001
**B. Dosimetric Parameters**					
Topogram length (mm)	386.55 ± 50.19	297.00	512.00	50.19	0.152
Topogram CTDIvol (mGy)	0.14 ± 0.01	0.10	0.14	0.01	0.331
Topogram DLP (mGy·cm)	5.15 ± 0.59	4.00	6.00	0.59	0.264
Diagnostic scan length (mm)	403.00 ± 29.84	341.00	445.00	29.84	0.001
CTDIvol (mGy)	1.33 ± 0.38	0.72	2.14	0.38	0.007
SSDE (mGy)	1.64 ± 0.33	1.10	2.44	0.33	0.096
DLP (mGy·cm)	50.35 ± 15.93	24.00	86.00	15.93	0.003
DLPss (mGy·cm)	61.93 ± 14.62	36.72	98.04	14.62	0.037

**Table 2:** Comparison of dose parameters between the LDCT group (*n* = 221) and the standard-dose chest CT group (*n* = 134) from the first study in the series [14].

Dose Parameters	LDCT (*n* = 221)	Standard CT (*n* = 134)	*p*	LDCT as % of Standard CT (=100% − Dose Reduction%)
CTDIvol (mGy)	1.33 ± 0.38	7.83 ± 2.92	<0.001	17.0%
SSDE (mGy)	1.64 ± 0.33	9.37 ± 2.31	<0.001	17.5%
DLP (mGy·cm)	50.35 ± 15.93	286.51 ± 99.82	<0.001	17.6%
DLPss (mGy·cm)	61.93 ± 14.62	343.90 ± 81.66	<0.001	18.0%
DLP of topogram (mGy·cm)	5.15 ± 0.59	5.28 ± 0.60	<0.05	97.5%
Topogram contribution to DLP (%)	10.23%	1.84%	<0.001	—

## Data Availability

The datasets used and analyzed during the current study are available from the corresponding author on reasonable request.

## Abbreviations

The following abbreviations are used in this manuscript:

ATCM	Attenuation-Based Tube Current Modulation
BMI	Body Mass Index
CNR	Contrast-to-Noise Ratio
COPD	Chronic Obstructive Pulmonary Disease
CT	Computed Tomography
CTDIvol	CT Dose Index Volume
Deff	Effective Diameter
DLIR	Deep Learning Image Reconstruction
DLP	Dose–Length Product
DLPss	Size-Specific Dose–Length Product
DRL	Diagnostic Reference Level
EID-CT	Energy-Integrating Detector CT
ED	Effective Dose
FBP	Filtered Back Projection
GGO	Ground Glass Opacity
IAEA	International Atomic Energy Agency
ICRP	International Commission on Radiological Protection
IR	Iterative Reconstruction
LDCT	Low-Dose Computed Tomography
NLST	National Lung Screening Trial
PCCT	Photon-Counting Computed Tomography
SD	Standard Deviation
SNR	Signal-to-Noise Ratio
SSDE	Size-Specific Dose Estimate
TCM	Tube Current Modulation
ULDCT	Ultra-Low-Dose Computed Tomography
UNSCEAR	United Nations Scientific Committee on the Effects of Atomic Radiation
